# Diverse Small Circular DNA Viruses Identified in an American Wigeon Fecal Sample

**DOI:** 10.3390/microorganisms12010196

**Published:** 2024-01-18

**Authors:** Diego Olivo, Anthony Khalifeh, Joy M. Custer, Simona Kraberger, Arvind Varsani

**Affiliations:** 1 Biodesign Center for Fundamental and Applied Microbiomics, Center for Evolution and Medicine, School of Life Sciences, Arizona State University, Tempe, AZ 85042, USA; daolivo@asu.edu (D.O.);; 2 Structural Biology Research Unit, Department of Integrative, Biomedical Sciences, University of Cape Town, Observatory, Cape Town 7925, South Africa

**Keywords:** *Cressdnaviricota*, *Microviridae*, *Mareca americana*, ssDNA virus, feces

## Abstract

American wigeons (*Mareca americana*) are waterfowls that are widely distributed throughout North America. Research of viruses associated with American wigeons has been limited to orthomyxoviruses, coronaviruses, and circoviruses. To address this poor knowledge of viruses associated with American wigeons, we undertook a pilot study to identify small circular DNA viruses in a fecal sample collected in January 2021 in the city of Tempe, Arizona (USA). We identified 64 diverse circular DNA viral genomes using a viral metagenomic workflow biased towards circular DNA viruses. Of these, 45 belong to the phylum *Cressdnaviricota* based on their replication-associated protein sequence, with 3 from the *Genomoviridae* family and the remaining 42 which currently cannot be assigned to any established virus group. It is most likely that these 45 viruses infect various organisms that are associated with their diet or environment. The remaining 19 virus genomes are part of the *Microviridae* family and likely associated with the gut enterobacteria of American wigeons.

## 1. Introduction

American wigeons (*Mareca americana*) are a widely distributed species of migratory waterfowl in North America [1]. *Mareca americana* is one of three species of wigeons, the other two being *M. penelope* and *M. sibilatrix* in the family Anatidae. American wigeons in the Pacific flyway overwinter in the southwest of North America, which includes the states of Arizona, California, and parts of Mexico. During the late spring/summer months, they migrate to parts of Canada and Alaska [2,3]. American wigeons feed in shallow bodies of freshwater (e.g., ponds, lakes, and marshes), and their diet primarily consists of plants and some insects [4]. American wigeons generally inhabit the same environments as other dabbling ducks, such as northern shovelers (*Spatula clypeata*), mallards (*Anas platyrhynchos*), green-winged teal (*Anas carolinensis*), northern pintails (*Anas acuta*), and gadwalls (*Mareca strepera*).

A significant amount of virology research has been undertaken on various waterfowl focusing on viruses in families *Adenoviridae*, *Astroviridae*, *Circoviridae*, *Coronaviridae*, *Flaviviridae*, *Herpesviridae*, *Orthomyxoviridae*, *Paramyxoviridae*, *Parvoviridae*, *Picornaviridae* and *Spinareoviridae*. However, within the context of American wigeons, most of the work has focused on orthomyxoviruses [5,6,7], coronaviruses [8], and circoviruses [9]. 

The *Cressdnaviricota* phylum is a recently established group of circular replication-encoding single-stranded (CRESS) DNA viruses [10]. Cressdnaviruses have relatively small genomes that contain at least two open reading frames (ORFs) that encode a conserved replication-associated protein (Rep) and a capsid protein (CP) [10]. Cressdnavirus families include *Amesuviridae*, *Bacilladnaviridae*, *Circoviridae*, *Genomoviridae*, *Geminiviridae*, *Nanoviridae*, *Naryaviridae*, *Nenyaviridae*, *Metaxyviridae*, *Redondoviridae*, *Smacoviridae*, and *Vilyaviridae* [10,11,12]. A significant number of cressdnaviruses have been identified via viral metagenomic studies [13,14,15,16,17,18,19,20,21,22]. 

The family *Genomoviridae* is divided into 10 genera based on the phylogeny of their Rep amino acid, i.e., *Gemycircularvirus*, *Gemyduguivirus*, *Gemygorvirus*, *Gemykibivirus*, *Gemykolovirus*, *Gemykrogvirus*, *Gemykronzavirus*, *Gemytondvirus*, *Gemytripvirus*, and *Gemyvongvirus* [23,24]. Genomoviruses have conserved regions in their Rep protein sequences that include the rolling circle replication (RCR) motifs, the gemini-like replication sequence (GRS), and Superfamily 3 (SF3) helicase motifs [25]. Although most genomoviruses have been found in various animal, plant, fungi, and environmental samples [24], there are confirmed hosts for two genomoviruses, i.e., Sclerotinia sclerotiorum [26] and Fusarium graminearum [27]. Genomoviruses are classified at a species level based on genome-wide pairwise identities. 

Microviruses are single-stranded DNA bacteriophages from the *Phixviricota* phylum with circular genome ranging from ~3 to 6 kb [28,29,30]. Microviruses have been previously identified in various samples across the globe [31]. Microviruses encode four structural proteins: major capsid protein F, major spike protein G, DNA pilot protein H, and DNA-binding protein J [32]. The *Microviridae* family is split into two subfamilies, *Gokushovirinae* and *Bullavirinae*. There are three genera (*Alphatrevirus*, *Gequatrovirus*, and *Sinsheimervirus*) in the *Bullavirinae* subfamily and four genera (*Bdellomicrovirus*, *Chlamydiamicrovirus*, *Enterogokushovirus*, and *Spiromicrovirus*) in the *Gokushovirinae* subfamily [29]. 

Given the limited information on viruses associated with American wigeons, we undertook a pilot metagenomic study to identify circular DNA viruses in a fecal sample collected in Tempe, Arizona (USA). We identified 3 genomoviruses, 42 unclassified cressdnaviruses, and 19 microviruses.

## 2. Materials and Methods

### 2.1. Fecal Sampling and High-Throughput Sequencing

An American wigeon fecal sample was collected on 13 January 2021 at Kiwanis Park, Tempe, Arizona, USA. The sample was collected using a sterile tongue depressor following a visual observation of an American wigeon defecating and then placed into a 2 mL tube. It was stored in a −20 °C freezer until processing. The fecal sample (1 g) was homogenized in 2 mL of SM buffer. The homogenate was centrifuged at 10,000× *g* for 10 min, and the supernatant was sequentially filtered through 0.45 µm and 0.2 µm syringe filters. In total, 200 µL of this filtrate was used to extract viral DNA using the High Pure Viral Nucleic Acid Kit (Roche, USA) following the manufacturer’s instructions. Circular DNA in the viral DNA extract was amplified using rolling circle amplification (RCA) with the Templiphi 100 amplification kit (GE Healthcare, USA). The RCA products were used to generate Illumina sequencing libraries using the DNA TrueSeq Nano kit, and they were sequenced on an Illumina Hiseq4000 sequencer (Illumina, USA) at Psomagen Inc. (USA). 

### 2.2. Sequence Assembly and Identification of Viral Contigs

The pair-end reads (2 × 150 nts) were trimmed using Trimmomatic v0.39 [33]. The resulting paired-end reads were then de novo assembled using MEGAHIT v1.2.9 [34], and contigs > 1000 nts in length were screened using BLASTx [35] against a viral RefSeq protein sequence database (release 207) for viral-like sequences. All contigs with terminal redundancy were determined to represent circular genomes. All circular genomes that appeared to be eukaryote-infecting viruses were annotated using ORFfinder (ncbi.nlm.nih.gov/orffinder/, accessed on 1 October 2023) coupled with manual checks. Prokaryote-infecting circular DNA viruses were annotated using VIBRANT [36]. 

Since multiple studies have identified some cressdnaviruses as reagent/kit contaminants [20,37,38,39,40], to identify any reagent-associated viruses or those misidentified as a result of barcode-hopping artifacts, we mapped all the reads from all the samples processed at the same time/run on the same lane to the virus genomes identified here using BBMap [41].

### 2.3. Analyses of Cressdnaviruses

A dataset was constructed of Rep sequences from representative (species-level) classified cressdnaviruses (*Bacillidnaviridae*, *Circoviridae*, *Geminiviridae*, *Genomoviridae*, *Metaxyviridae*, *Nanoviridae*, *Naryaviridae*, *Nenyaviridae*, *Redondoviridae*, *Smacoviridae*, and *Vilyaviridae*), alphasatellites, all CRESS Groups 1–6 [42,43], and all unclassified cressdnaviruses. This dataset, together with Reps of the cressdnaviruses from this study, was used to determine putative family-level grouping with a sequence similarity network (SSN) using EFI-EST [44] with a similarity score of 60. This threshold allows for putative family-level clustering for cressdnaviruses [13,17,19,45,46,47,48]. The SSN of the resulting Rep amino acid sequences was visualized in Cytoscape v3.8.2 [49] with an organic layout visualization option. 

We extracted Rep protein sequences from the representative Rep sequence dataset used for SSN analysis that cluster with those from this study, as well as those from the established viral cressdnavirus families and CRESS Groups 1–6. These were collectively aligned with MAFFT v7.113 [50], and the resulting alignment was trimmed using TrimAL with a gap threshold of 0.2 [51]. A maximum likelihood phylogenetic tree was constructed using IQTree v2.1.3 [52] with a Q.pfam + F + G4 substitution model identified as the best-fit model and with approximate likelihood ratio test (aLRT) branch support [53] inferred from the trimmed alignment. The maximum likelihood phylogenetic tree was visualized with iTOL v6 [54].

The Rep amino acid sequences form the *Genomoviridae* family and unclassified cressdnavirus clusters (CRESSV2, CRESSV6, and Clusters A–Q) were individually aligned using MAFFT v7.113 AUTO mode [50] with appropriate outgroups based on the large Rep phylogenetic tree. Cluster-level alignments were used to determine the best-fit amino acid substitution model using ProtTest3 [55], and maximum likelihood trees were inferred with these models and PhyML3 [56]. In the resulting trees, branches with <0.80 aLRT branch support [53] were collapsed in TreeGraph2 [57]. All pairwise identities were determined using SDTv1.2 [58].

### 2.4. Analyses of Microviruses

Major capsid protein (MCP) sequences were extracted from the genome sequences of microviruses and assembled into a dataset that contained 3641 known MCP sequences. The MCP sequences were translated and aligned with those from the study using the MAFFT v7.113 AUTO mode [50]. The resulting alignments were trimmed using TrimAl v1.2 with the gappyout option [51]. The trimmed alignment was used to infer a maximum likelihood phylogenetic tree IQTree v2.1.3 using the best-fit model [52] and visualized with iTOL v6 [59].

## 3. Results and Discussion

### 3.1. Identification of Viral Genomes

The de novo assemblies resulted in 3538 contigs with a size range of 200–66,203 nts. Of these, 1228 were >1000 nts. Of these, 672 were identified to be viral-like based on BLASTx analysis representing viruses in phyla *Cressdnaviricota* (*n* = 82), *Hofneiviricota* (*n* = 10), *Nucleocytoviricota* (*n* = 45), *Phixviricota* (*n* = 24) and *Uroviricota* (*n* = 511). Of all of these, 64 contigs with similarities to viruses in *Cressdnaviricota* (*n* = 45) and *Phixviricota* (*n* = 19) were identified to have terminal redundancies and thus determined as ones representing complete circular genomes. No raw reads mapping to these contigs were found in any of the other sample libraries processed at the same time in the lab and run on the same flow cell based on our mapping analysis using BBMap [41]. 

**Figure 1:**
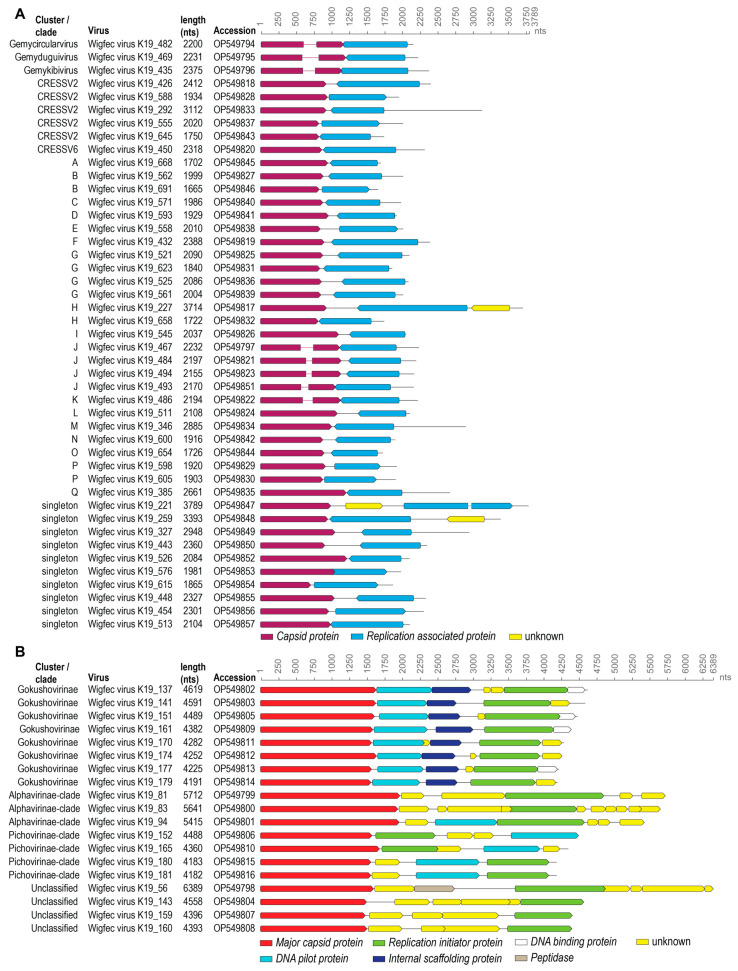
Summary of the genomes of cressdnaviruses (**A**) and microviruses (**B**) identified from the American wigeon fecal sample. Circular genomes are shown in a linear representation.

**Figure 2:**
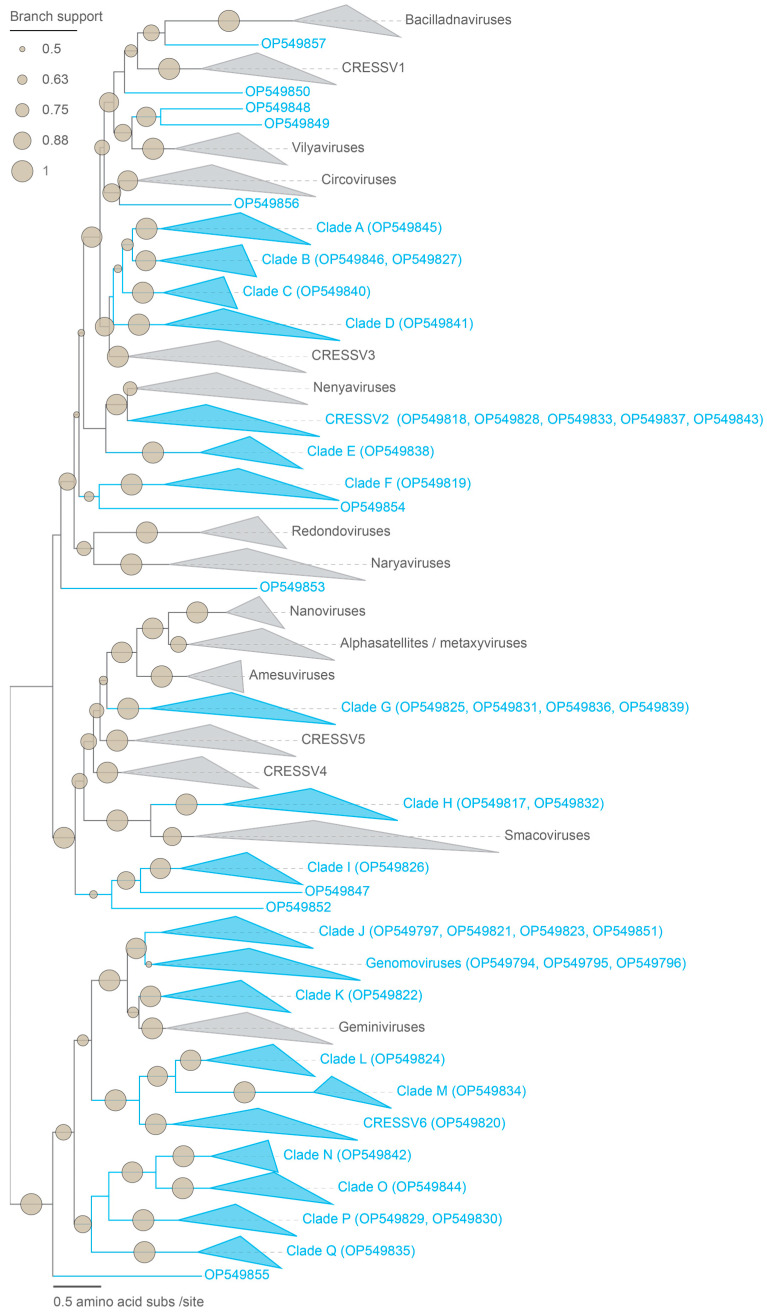
The Rep amino acid maximum likelihood phylogenetic tree inferred with IQTree2 (Minh et al., 2020) [52] with Q.pfam + F + G4 substitution model identified as the best-fit model for the viruses in the *Cressdnaviricota* phylum. The family-level clustering for unclassified CRESS groups was determined by sequence similarity networks (SSN) of the amino acid sequences of the cressdnavirus Rep with a sequence similarity score of 60 using EFI-EST [44] and visualized with Cytoscape v3.8.2 [49]. The Reps identified from this study are shown in blue and are grouped into the *Genomoviridae* family, 20 family-level clusters (CRESSV2, CRESSV6, A–Q), and 10 singletons.

For this study, we focus on the complete genomes (Figure 1). Three of the 45 cressdnaviruses are part of the family *Genomoviridae* and the rest cluster (based on the Rep sequence similarity network) with sequences of unclassified cressdnaviruses. Collectively, the Reps of these cressdnaviruses are part of 1 classified cluster (genomoviruses) and 19 unclassified clusters (CRESSV2, CRESSV6, clusters A–Q), and 10 are singletons (Figure 2). The 19 phixviruses are part of the family *Microviridae*. 

A summary of the BLASTn [35] analysis of the genomes identified here is provided in Table 1. With the exception of three viruses, i.e., wigfec virus K19_469 (OP549795), wigfec virus K19_561 (OP549839) and wigfec virus K19_141 (OP549803) which share >70% pairwise identity with >80% genome coverage, all others are relatively diverse.

### 3.2. Genomoviruses

**Figure 3:**
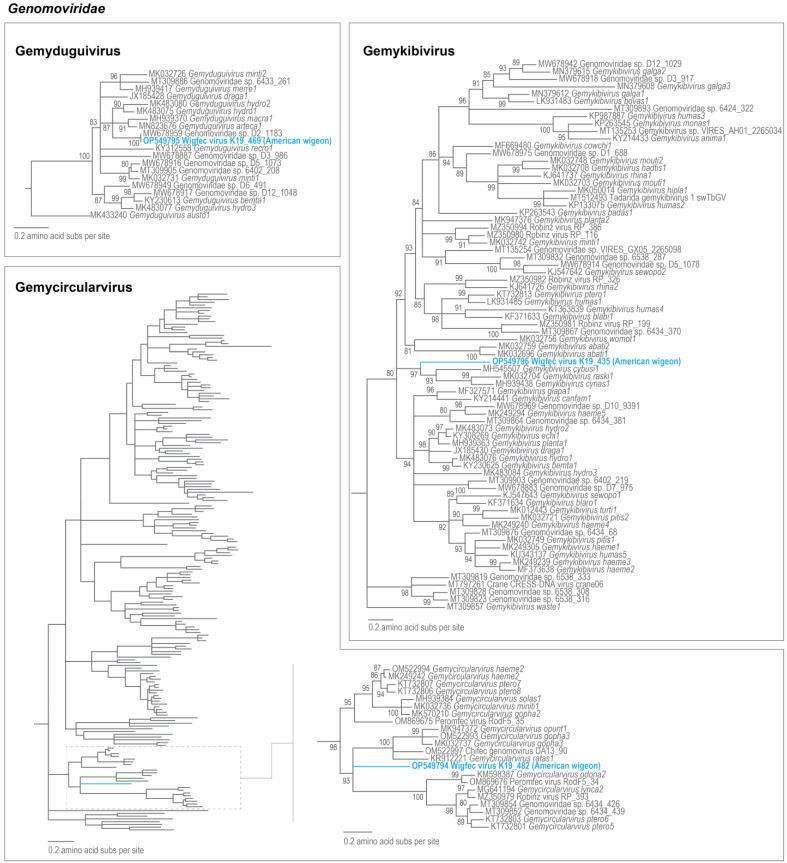
Maximum likelihood phylogenetic relationship of the Rep protein sequences of representative sequences (species-level) of viruses in genera *Gemyduguivirus*, *Gemykibivirus*, and *Gemycircularvirus*. The maximum likelihood phylogenetic tree was inferred using PhyML 3 [56] and rooted with Rep sequences of geminiviruses with LG + G + I as best-fit models determined using ProtTest 3 [55]. All sequences from this study are highlighted in blue font, and for gemycircularviruses, a zoomed-in section of phylogeny is shown.

The genomoviruses identified in this study range in size from 2200 to 2375 nts and encode a CP and a Rep in an ambisense orientation [24]. The three genomoviruses identified in this study belong to three different genera with wigfec virus K19_435 (OP549796) in *Gemykibivirus*, wigfec virus K19_469 (OP549795) in *Gemyduguivirus* and wigfec virus K19_482 (OP549794) in *Gemycircularvirus* (Figure 3). The conserved rolling circle replication motif (RCR), geminivirus Rep-like sequences (GRS), and Superfamily 3 (SF3) helicase motifs are present in all the Reps of wigfec genomoviruses (Table 2).

Wigfec virus K19_469 (OP549795) is most similar to *Genomoviridae* sp. D2_1183 (MW678959), isolated from dust particles in Arizona [60], which is not classified at a species level sharing 98% genome-wide nucleotide pairwise identity and 100% Rep amino acid identity (Table 3). Given this virus was also detected in Arizona, it may be that it infects a commonly detected fungus in Arizona. Wigfec virus K19_435 (OP549796) is most similar to Cybaeus spider-associated circular virus 2 BC_I1644B_C3 (MH545507) [61] which belongs to species *Gemykibivirus cybusi1*, sharing 51% genome-wide nucleotide pairwise identity. Its Rep shares 60% amino acid identity and clusters with other members of species *Gemykibivirus cynas1* and *Gemykibivirus raski1* (Figure 3). Wigfec virus K19_482 (OP549794) is most similar to gemycircularvirus gemy-ch-rat1 (KR912221), identified from a rat [62], which is part of species *Gemycircularvirus ratas1*, sharing 51% genome-wide nucleotide pairwise identity and 38% Rep amino acid identity (Table 3). 

Wigfec virus K19_435, wigfec virus K19_482, and wigfec virus K19_469 with *Genomoviridae* sp. D2_1183 represent three new species based on the previously established 78% genome-wide pairwise identity species demarcation threshold for genomoviruses [23]. All the three genomoviruses identified here are likely fungal-infecting viruses based on what is known of two of the fungal hosts (*Sclerotinia sclerotiorum* and *Fusarium graminearum*) [26,27], specific genomviruses in species *Gemycircularvirus sclero1* and *Gemytripvirus fugra1* [24].

### 3.3. Unclassified Cressdnaviruses

Forty-two cressdnaviruses (size range 1665–3789 nts) could not be assigned to any established cressdnavirus family (Figure 1 and Figure 2). Based on SSN analysis, the Reps of 10 cressdnaviruses are singletons, and 32 cluster with other known Reps within 19 unique clusters (Figure 2). This highlights the diversity of these cressdnaviruses within a single fecal sample. Rep amino acid phylogenetic analysis for each cluster with >2 sequences is undertaken. In the Reps of all these 42 cressdnaviruses, we identify the conserved RCR and SF3 helicase motifs (Table 2). Additionally, in the Reps of wigfec virus K19_467 (OP549797), wigfec virus K19_484 (OP549821), wigfec virus K19_494 (OP549823), and wigfec virus K19_493 (OP549851), which are all part of Cluster J, and wigfec virus K19_486 (OP549822) which is part of Cluster K, we identified a GRS domain (Table 2). The GRS domain in the Rep of wigfec virus K19_486 appears to have a five-residue insertion (DGTVY) (Table 2).

**Figure 4:**
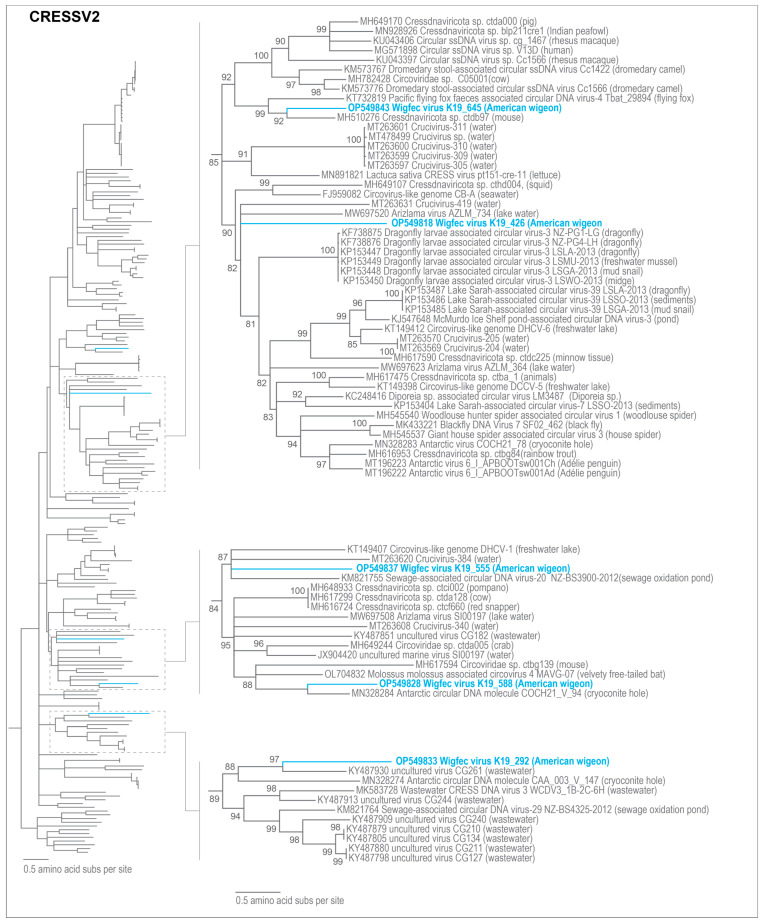
Maximum likelihood phylogenetic relationship of the Rep protein sequences of unclassified cressdnaviruses in clusters CRESSV2. The maximum likelihood phylogenetic tree was inferred using PhyML 3 [56] with VT + I + G as best-fit model determined using ProtTest 3 [55] and rooted with Rep sequences from the CRESSV5 cluster. Sections of phylogeny are zoomed in to show the details in relation to the Reps from this study of the viruses that are part of the CRESSV2 cluster. All sequences from this study are highlighted in blue font.

CRESSV1-6 have previously been described as unique family level groupings [43]. Five of the viruses identified in this study (wigfec virus K19_426 (OP549818), wigfec virus K19_588 (OP549828), wigfec virus K19_292 (OP549833), wigfec virus K19_555 (OP549837) and wigfec virus K19_645 (OP549843) are part of CRESSV2 (Figure 4), and they share ~32–40% amino acid identity and <57% amino acid identity with the Reps of all other viruses in cluster CRESSV2 and are distributed throughout the CRESSV2 Rep phylogeny (Figure 4). 

**Figure 5:**
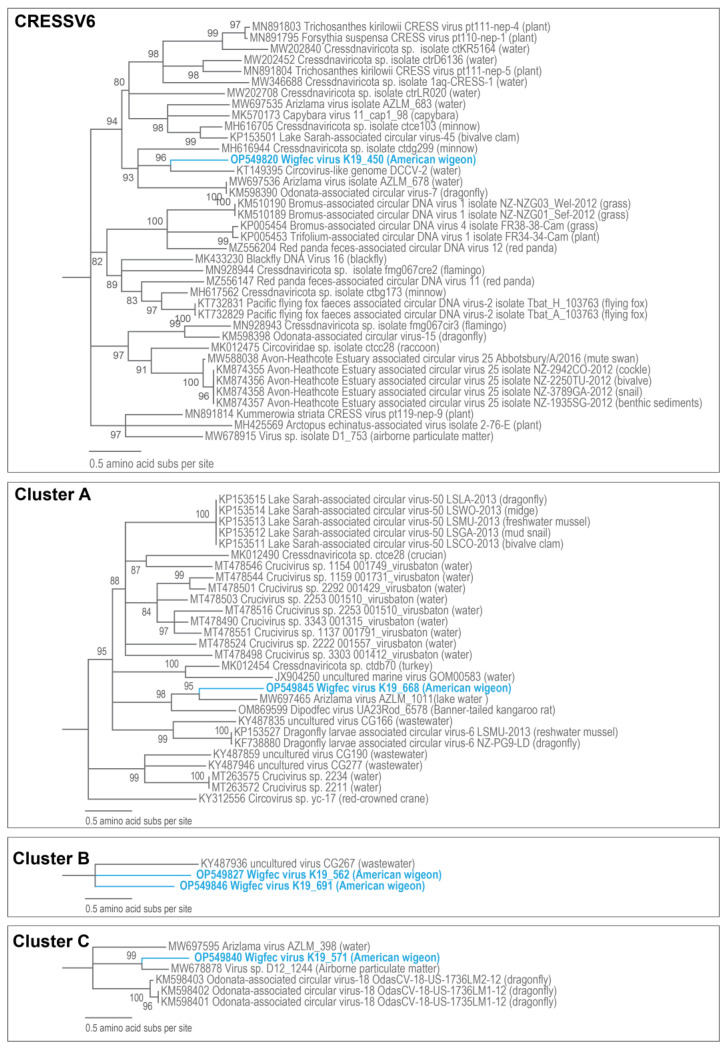
Maximum likelihood phylogenetic relationship of the Rep protein sequences of unclassified cressdnaviruses in Clusters CRESSV6 (rooted with redondovirus Rep sequences) and Clusters A, B, and C (rooted with CRESSV5 Rep sequences). The maximum likelihood phylogenetic trees of each cluster were inferred using PhyML 3 [56] with LG + I + G for the CRESSV2 cluster and LG + I + G + F for Clusters A, B, and C as best-fit models determined using ProtTest 3 [55]. All sequences from this study are highlighted in blue font.

The Reps of wigfec virus K19_426, wigfec virus K19_588, wigfec virus K19_292, wigfec virus K19_555, and wigfec virus K19_645 are most similar to those of *Diporeia* sp. associated circular virus LM3487 (KC248416) [63], Antarctic circular DNA molecule COCH21_V_94 (MN328284) [64], uncultured virus CG261 (KY487930) [65], sewage-associated circular DNA and virus-20 NZ-BS3900-2012 (KM821755) [66], and *Cressdnaviricota* sp. ctdb97 (MH510276) [20], sharing 46%, 57%, 46%, 53%, and 56% amino acid identity, respectively (Table 3). Wigfec virus K19_450 (OP549820) is part of CRESSV6 (Figure 5). The Rep of wigfec virus K19_450 (OP549820) shares a pairwise amino acid identity of 49.4% with that of Circovirus-like DCCV-2 (KT149395) identified from a freshwater lake in China and phylogenetically forms a clade with it, as well (Figure 5, Table 3). 

**Figure 6:**
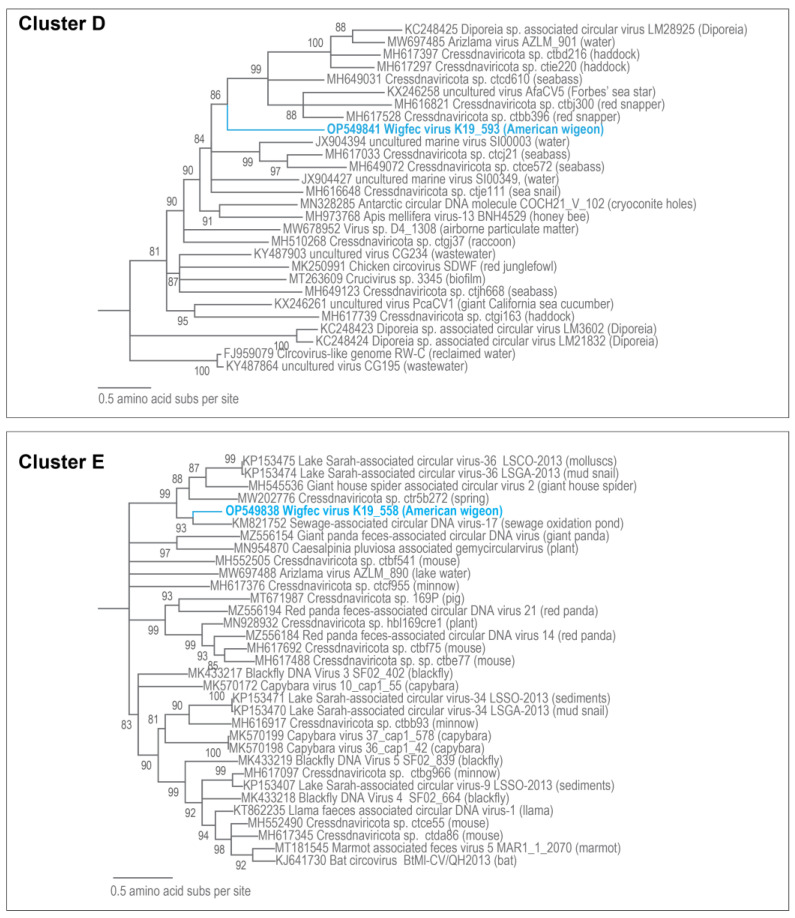
Maximum likelihood phylogenetic relationship of the Rep protein sequences of unclassified cressdnaviruses in Clusters D and E (both rooted with CRESSV5 Rep sequences). The maximum likelihood phylogenetic trees of each cluster were inferred using PhyML 3 [56] with LG + I + G + F for Cluster D cluster and RtRev + I + G + F for Cluster E as best-fit models determined using ProtTest 3 [55]. All sequences from this study are highlighted in blue font.

**Figure 7:**
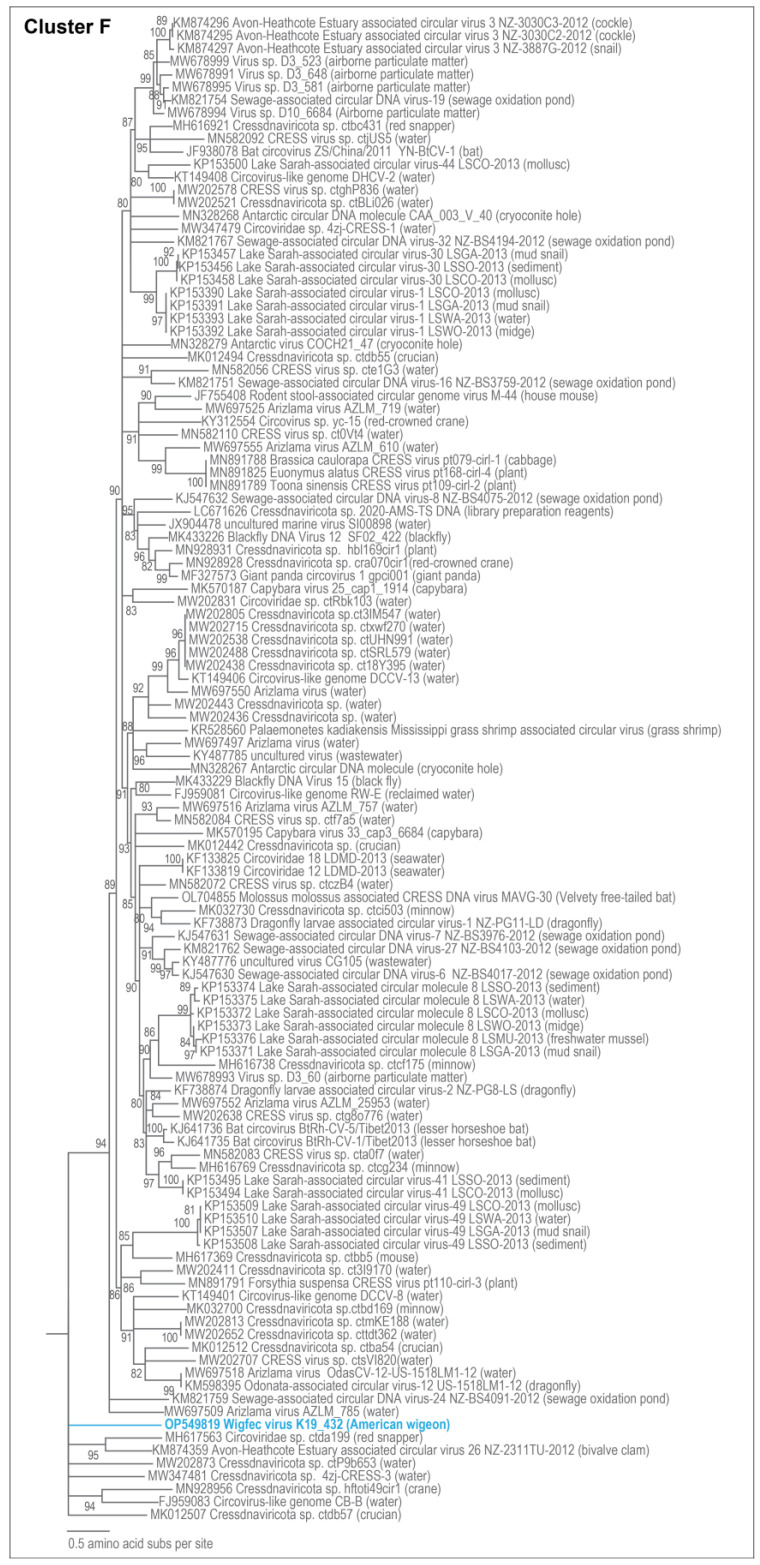
Maximum likelihood phylogenetic relationship of the Rep protein sequences of unclassified cressdnaviruses in Cluster F. The maximum likelihood phylogenetic tree was inferred using PhyML 3 [56] with LG + I + G as best-fit model determined using ProtTest 3 [55] and rooted with Rep sequences from the redondoviruses. The sequence from this study is highlighted in blue font.

The Rep of wigfec virus K19_668 (OP549845) is part of Cluster A and shares 51% amino acid identity and clustering with Arizlama virus isolate AZLM_1011(MW697465), which was detected in a lake sample from Arizona (Figure 5). The Reps of wigfec virus K19_562 (OP549827) and wigfec virus K19_691 (OP549846) cluster and that of uncultured virus CG267 (KY487936) [65] share < 44% amino acid identity (Figure 5, Table 3). The Rep of wigfec virus K19_571 (OP549840) clusters with the Reps of five viruses in Cluster C share ~46–64% amino acid identity, and it is most closely related to that of *Virus* sp. isolate D12_1244 (MW678878) [60]. The Reps of wigfec virus K19_593 (OP549841), wigfec virus K19_558 (OP549838), and wigfec virus K19_432 (OP549819) are part of Clusters D, E, and F, respectively (Figure 6 and Figure 7). Their Reps share the highest similarity of 45%, 72%, and 51% amino acid identity with Reps of *Cressdnaviricota* sp. ctcd610 (MH649031) [20], Sewage-associated circular DNA virus-17 (KM821752) and Avon-Heathcote Estuary-associated circular virus 26 NZ-2311TU-2012 (KM874359) [14], respectively (Table 3).

**Figure 8:**
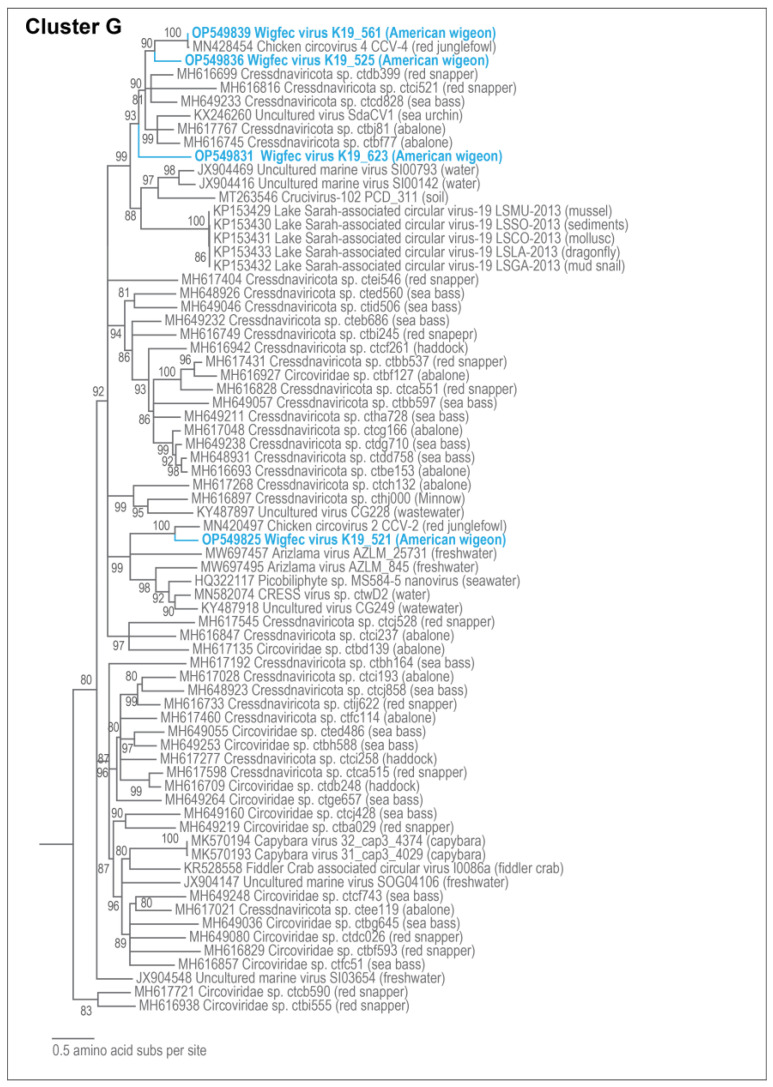
Maximum likelihood phylogenetic relationship of the Rep protein sequences of unclassified cressdnaviruses in Cluster G. The maximum likelihood phylogenetic tree was inferred using PhyML 3 [56] with RtRev + I + G + F as best-fit model determined using ProtTest 3 [55] and rooted with Rep sequences from the redondoviruses. All sequences from this study are highlighted in blue font.

In Cluster G, the genome of wigfec virus K19_561 (OP549839) shares ~90% similarity with the genome of Chicken circovirus 4 CCV-4 (MN428454) identified in the stomach of a red junglefowl (*Gallus gallus*), from southeast Asia [67] (Table 3). Their Reps share 98.6% amino acid identity. This virus is the only unclassified cressdnavirus that has high similarity to a previously identified virus. Furthermore, the Rep of wigfec virus K19_521 (OP549825) shares ~63% with chicken circovirus 2 CCV-2 (MN420497), also from red junglefowl [67] (Table 3). The Rep of wigfec virus K19_525 (OP549836) clusters with the Reps of wigfec virus K19_561 and Chicken circovirus 4 CCV-4, sharing ~59% amino acid identity (Figure 8). Given that several of these circovirus-like genomes have been detected in two bird species, it may be that this is an avian virus or infects an organism that is commonly associated with avian species. 

**Figure 9:**
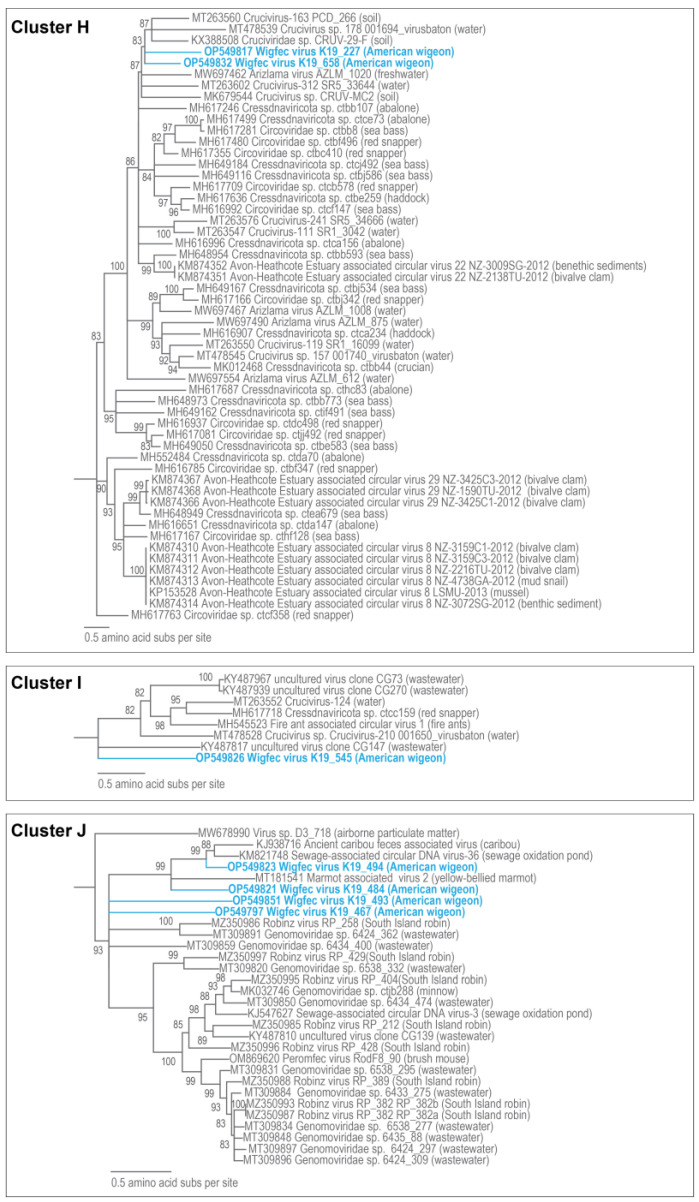
Maximum likelihood phylogenetic relationship of the Rep protein sequences of unclassified cressdnaviruses in Clusters H, I, and J (all rooted with redondovirus Rep sequences). The maximum likelihood phylogenetic trees of each cluster were inferred using PhyML 3 [56] with RtRev + I + G + F for Cluster H, VT + I + G + F for Cluster I, and LG + I + G + F for Cluster J as best-fit models determined using ProtTest 3 [55]. All sequences from this study are highlighted in blue font.

The Rep of wigfec virus K19_623 (OP549831) in Cluster G shares 54% amino acid identity with the Rep of *Cressdnaviricota* sp. ctcd828 (MH649233) from seabass tissue [20]. Wigfec virus K19_227 (OP549817) and wigfec virus K19_658 (OP549832) are part of Cluster H (Figure 9), and their Reps share ~48% amino acid identity and ~49 and 53% amino acid identity with *Cressdnaviricota* sp. ctbb593 (MH648954) from seabass tissue and *Cressdnaviricota* sp. ctca156 (MH616996) from abalone tissue [20] (Table 3). The Rep of wigfec virus K19_545 (OP549826) in Cluster I shares ~43% amino acid identity with the Rep of Crucivirus-124 BS_313 (MT263552) from a water sample in New Zealand [15] (Figure 9, Table 3). 

The Reps of wigfec virus K19_467 (OP549797), wigfec virus K19_484 (OP549821), wigfec virus K19_494 (OP549823), and wigfec virus K19_493 (OP549851) are part of Cluster J, sharing 32–54% amino acid identity and 46, 53, 65 and 37% amino acid identities with the Reps of *Genomoviridae* sp. 6434_400 (MT309859), Ancient caribou feces-associated virus (KJ938716) [68], Sewage-associated circular DNA virus-36 NZ-BS3974-2012 (KM821748) [66], and *Genomoviridae* sp. 6538_332 (MT309820), respectively (Table 3). Wigfec virus K19_486 (OP549822), a member of Cluster K, encodes a Rep that shares 47% amino acid identity with that of *Genomoviridae* sp. 6538_302 (MT309829) from wastewater. The Reps of the members in Clusters J and K all have a GRS domain (Table 2), and, although some are named “Genomoviridae”, they belong to an outgroup most closely related to genomoviruses and geminiviruses (Figure 2).

**Figure 10:**
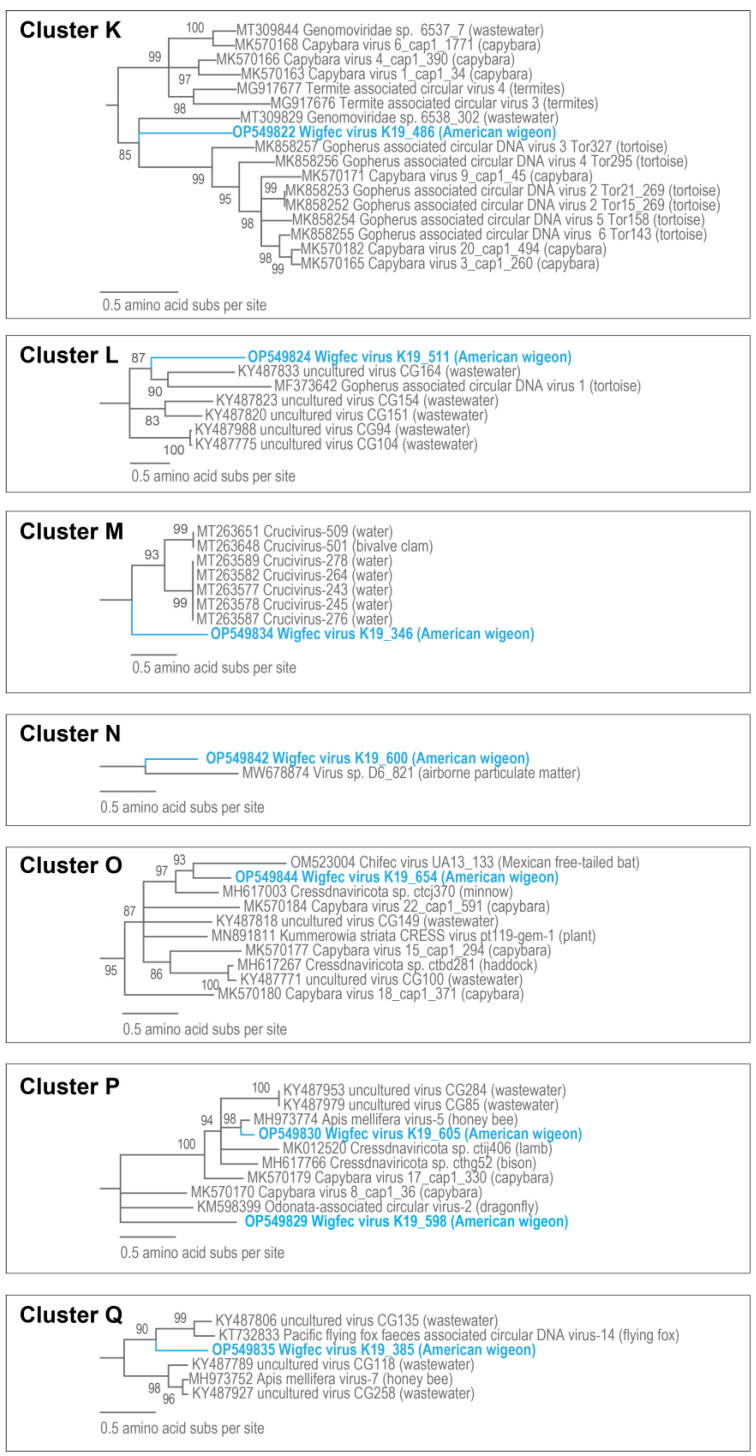
Maximum likelihood phylogenetic relationship of the Rep protein sequences of unclassified cressdnaviruses in Clusters K, L, M, N, O, P, and Q. The maximum likelihood phylogenetic trees of each cluster with Reps of geminiviruses for Cluster K, CRESSV6 for Clusters L and M, and redondoviruses for Clusters O, P, and Q, and rooting sequences were inferred using PhyML 3 [56] with LG + I + G + F (Cluster K), LG + I + G (Clusters M and N), and RtRev + I + G + F (Clusters O, P, and Q) as best-fit models determined using ProtTest 3 [55]. All sequences from this study are highlighted in blue font.

The Reps of wigfec virus K19_511 (OP549824) and uncultured virus clone CG104 (KY487775) [65] share 41% amino acid pairwise identity in Cluster L (Figure 10), and that of wigfec virus K19_346 (OP549834) shares 47% with that of Crucivirus-243 SR3_42497 (MT263577) [15] in Cluster M (Table 3). Cluster N is made of two Rep sequences, i.e., wigfec virus K19_600 (OP549842) and *Virus* sp. isolate D6_821 (MW678874) [60], sharing ~47% amino acid identity. In Cluster O, the Rep of wigfec virus K19_654 (OP549844) shares ~49 and 59% amino acid identity with the Reps of chifec virus UA13_133 (OM523004) [46] from a Mexican free tailed bat and *Cressdnaviricota* sp. ctcj370 (MH617003) from minnow tissue [20] (Figure 10, Table 3). The Reps of wigfec virus K19_598 (OP549829) and wigfec virus K19_605 (OP549830) in Cluster P (Figure 10) share 46% amino acid identity amongst them and 46% and 86% with those of Capybara virus 8_cap1_36 (MK570170) [45] and Apis mellifera virus-5 BNH861 (MH973774) [16], respectively (Table 3). Wigfec virus K19_385 (OP549835) is part of Cluster Q, and its Rep shares 52% amino acid identity with the Rep of uncultured virus CG135 (KY487806) [65] (Table 3). 

The 10 singletons, wigfec virus K19_221 (OP549847), wigfec virus K19_259 (OP549848), wigfec virus K19_327 (OP549849), wigfec virus K19_443 (OP549850), wigfec virus K19_526 (OP549852), wigfec virus K19_576 (OP549853), wigfec virus K19_615 (OP549854), wigfec virus K19_448 (OP549855), wigfec virus K19_454 (OP549856), and wigfec virus K19_513 (OP549857), share 26–41% Rep amino acid identity with the best hits based on BLASTp analysis (Table 3).

### 3.4. Microviruses

**Figure 11:**
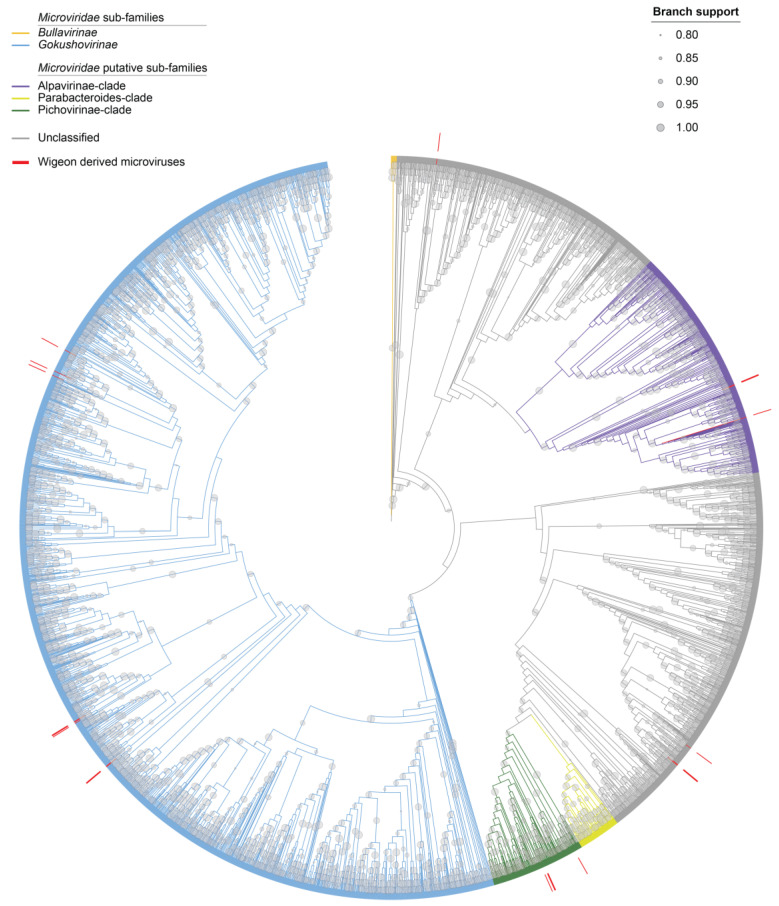
Maximum likelihood cladogram of the major capsid protein (MCP) sequences from members of the *Microviridae* family inferred using IQTree2 with LG + F + G4 (Minh et al., 2020) [52] determined as the best-fit amino acid substitution model. Branches are shown with branch support > 0.8 aLRT. Sub-families *Bullavirinae*, *Gokushovirinae*, putative families Alpavirinae, Parabacteroides, and Pichovirinae are shown in different-colored clades.

We identified 19 microviruses that range in size from 4182 to 6389 nts. All of the 19 microviruses encode a major capsid protein (MCP) and a replication initiator protein. The MCP phylogeny reveals that those from this study are broadly distributed across several clades, with eight in the subfamily of Gokushovirinae, three in proposed putative sub-family clade Alpavirinae, and four in the Pichovirinae (Figure 1 and Figure 11) [69]. Four of the identified microviruses fall outside of these (Figure 1 and Figure 11). The MCPs of these viruses in general share 38–77% highest amino acid identity with those of microviruses identified from various environments (Table 4). These microviruses likely infect the gut enterobacteria of the American wigeon, and they all represent new species based on the 95% species threshold used for bacteriophage, as these genomes share < 88% genome-wide identity with all other microvirus genomes in GenBank.

## 4. Conclusions

American wigeons play a vital ecological role in wetland ecosystems across North America. These birds travel hundreds of kilometers during their migration seasons and can provide insight into viral diversity due to their interactions across different habitats. We identified 42 unclassified cressdnavirus, 3 genomovirus, and 19 microvirus genomes through our non-invasive fecal sampling approach from one sample. The unclassified cressdnaviruses identified from this study are diverse. The three members of the Genomoviridae family are part of three different genera: *Gemykibivirus*, *Gemyduguivirus*, and *Gemycircularvirus*. These genomoviruses most likely infect fungi associated with American wigeons; however, in general, little is known about their host range. In total, 10 cressdnaviruses are singletons, and 32 cluster into 20 family-level groups. In addition, 3 cressdnaviruses, wigfec virus K19_521, wigfec virus K19_467, and wigfec virus K19_561, are most similar to genomes detected from avian samples, and wigfec virus K19_469 is most similar to a Gemyduguivirus from airborne dust particles; however, the rest are diverse viruses. In general, all these 42 unclassified cressdnaviruses each likely represent at least 40 new species of viruses, as they share < 80% genome-wide identity with other virus genomes in GenBank. The 19 microviruses we identified most likely infect the gut microbiota of the American wigeon and these all represent 19 new species. This pilot study highlights the diverse viral community within just a single fecal sample of an American wigeon. Although we cannot determine whether any of the eukaryote-infecting viruses we identified in this study infect the American wigeon, they expand our knowledge on diversity of ssDNA viruses, and with more studies, we will be able to start understanding the ecology of these viruses.

## Figures and Tables

**Table 1:** Summary of the BLASTn analysis of the virus genomes identified in this study showing top hits with genome coverage, e-value, and percentage identity. Those with—indicate they had no BLASTn hit.

		Top BLASTn Hit				
Cluster	Accession	Virus	Hit Coverage	E Value	% Identity	Hit Accession
Genomovirus	OP549794	Genomoviridae sp. D1_734	38%	1 × 10^−83^	75.51%	MW678940
Genomovirus	OP549795	Genomoviridae sp. D2_1183	100%	0	97.94%	MW678959
Genomovirus	OP549796	Plant-associated genomovirus 2 GnOP3_BA764	27%	6 × 10^−44^	73.26%	MH939416
CRESSV2	OP549818	Circoviridae sp. CN13_L19_1119	22%	1 × 10^−20^	65.49%	MT208232
CRESSV2	OP549828	Cressdnaviricota sp. Miresoil virus 170	12%	2 × 10^−42^	77.54%	OM154655
CRESSV2	OP549833	Circoviridae sp. CN13_L15_2544	13%	2 × 10^−38^	69.90%	MT203404
CRESSV2	OP549837	Circoviridae sp. CN13_L18_554	5%	2 × 10^−17^	79.49%	MT207945
CRESSV2	OP549843	Circoviridae sp. CN13_L03_572	45%	2 × 10^−138^	75.24%	MT201433
CRESSV6	OP549820	*CRESS virus* sp. ctWTK558	64%	7 × 10^−88^	65.79%	MW202429
A	OP549845	Banfec virus 6 V16_S08b	15%	1 × 10^−37^	73.98%	OQ599930
B	OP549827	Cressdnaviricota sp. Miresoil virus 407	5%	4 × 10^−08^	74.55%	OM154420
B	OP549846	Cressdnaviricota sp Miresoil virus 476	14%	2 × 10^−29^	73.53%	OM154360
C	OP549840	Virus sp. D12_1244	53%	1 × 10^−45^	65.68%	MW678878
D	OP549841	Cressdnaviricota sp. ctje111	14%	2 × 10^−17^	68.53%	MH616648
E	OP549838	Sewage-associated circular DNA virus-17 NZ-BS4236-2012	37%	5 × 10^−120^	73.39%	KM821752
F	OP549819	Avon-Heathcote Estuary-associated circular virus 3 NZ-3887G-2012	8%	1 × 10^−15^	71.35%	KM874297
G	OP549825	Chicken circovirus 2 CCV-2	37%	7 × 10^−81^	69.69%	MN420497
G	OP549831	Chicken circovirus 4 CCV-4	38%	1 × 10^−38^	68.44%	MN428454
G	OP549836	Chicken circovirus 4 CCV-4	30%	4 × 10^−46^	67.50%	MN428454
G	OP549839	Chicken circovirus 4 CCV-4	100%	0	90.87%	MN428454
H	OP549817	Crucivirus-93 GP1_93001	9%	3 × 10^−19^	67.64%	MT263542
H	OP549832	Cruciviridae sp. CRUV-29-F	31%	7 × 10^−29^	67.35%	KX388508
I	OP549826	Cressdnaviricota sp. Miresoil virus 407	5%	4 × 10^−08^	74.55%	OM154420
J	OP549797	Capybara virus 5_cap1_460	10%	8 × 10^−17^	69.80%	MK570167
J	OP549821	Egret CRESS-DNA virus egret03	49%	9 × 10^−99^	68.37%	MT797255
J	OP549823	Sewage-associated circular DNA virus-36 NZ-BS3974-2012	48%	9 × 10^−156^	72.43%	KM821748
J	OP549851	Trichosanthes kirilowii geminiviridae pt111-gem-5	6%	9 × 10^−10^	72.79%	MN823663
K	OP549822	Gopherus-associated circular DNA virus 3 Tor327	14%	2 × 10^−18^	68.63%	MK858257
L	OP549824	-	-	-	-	-
M	OP549834	Circoviridae sp. CN55_L18_367	24%	6 × 10^−33^	66.39%	MT207798
N	OP549842	Cressdnaviricota sp. Miresoil virus 418	45%	4 × 10^−90^	69.28%	OM154411
O	OP549844	Capybara virus 15_cap1_294	10%	7 × 10^−10^	69.66%	MK570177
P	OP549829	Cressdnaviricota sp. Miresoil virus 170	12%	2 × 10^−42^	77.54%	OM154655
P	OP549830	Apis mellifera virus-5 BNH861	54%	0	79.92%	MH973774
Q	OP549835	Uncultured virus clone CG135	33%	2 × 10^−44^	66.06%	KY487806
Singleton	OP549847	Crucivirus sp. Crucivirus-391 001074_virusbaton	7%	1 × 10^−17^	68.86%	MT478484
Singleton	OP549848	-	-	-	-	-
Singleton	OP549849	Circoviridae sp. CN3_L17_554	1%	0.004	92.31%	MT206222
Singleton	OP549850	Cressdnaviricota sp. Miresoil virus 557	16%	9 × 10^−23^	68.20%	OM154279
Singleton	OP549852	-	-	-	-	-
Singleton	OP549853	-	-	-	-	-
Singleton	OP549854	-	-	-	-	-
Singleton	OP549855	Cressdnaviricota sp. Miresoil virus 386	8%	0.039	68.02%	OM154441
Singleton	OP549856	Cressdnaviricota sp. Miresoil virus 388	3%	0.039	75.32%	OM154439
Singleton	OP549857	-	-	-	-	-
Microvirus	OP549798	Tortoise microvirus 72_SP_41	44%	1 × 10^−115^	72.38%	MK765625
Microvirus	OP549799	Microviridae sp. ctdg534	15%	1 × 10^−38^	67.76%	MH616940
Microvirus	OP549800	Microviridae sp. ctdg534	20%	6 × 10^−61^	69.09%	MH616940
Microvirus	OP549801	Microviridae sp. ctci549	54%	7 × 10^−162^	71.14%	MH617187
Microvirus	OP549802	Apis mellifera-associated microvirus 29 INH_SP_235	13%	1 × 10^−38^	68.86%	MH992203
Microvirus	OP549803	Microviridae sp. CN7_L19_255	81%	0	70.73%	MT208143
Microvirus	OP549804	Microviridae sp. Dog06	34%	3 × 10^−172^	69.41%	MG883726
Microvirus	OP549805	Arizlama microvirus AZLM_329	52%	6 × 10^−136^	74.36%	MW697640
Microvirus	OP549806	Microviridae sp. ctba71	33%	5 × 10^−68^	69.33%	MH616766
Microvirus	OP549807	Arizlama microvirus AZLM_380	31%	7 × 10^−104^	72.45%	MW697604
Microvirus	OP549808	Microvirus sp. 6433_74	31%	1 × 10^−106^	72.40%	MT310103
Microvirus	OP549809	Arizlama microvirus AZLM_274	35%	4 × 10^−170^	69.40%	MW697684
Microvirus	OP549810	Microviridae sp. SD_MC_24	12%	2 × 10^−33^	67.44%	MH572460
Microvirus	OP549811	Microvirus sp. BS1_385	65%	3 × 10^−146^	69.91%	MT309971
Microvirus	OP549812	Robinz microvirus RP_38	3%	1 × 10^−11^	73.91%	MZ364230
Microvirus	OP549813	Microviridae sp. ctbi780	25%	6 × 10^−98^	70.78%	MH622931
Microvirus	OP549814	Microvirus sp. BS1_385	62%	5 × 10^−124^	72.73%	MT309971
Microvirus	OP549815	Tortoise microvirus 93_SP_131	20%	9 × 10^−45^	66.97%	MK765646
Microvirus	OP549816	Tortoise microvirus 93_SP_131	22%	7 × 10^−40^	66.06%	MK765646

**Table 2:** Summary of the RCR and SF3 helicase motifs identified in the Reps of the cressdnaviruses from this study.

Cluster	Accession	Motif I	Motif II	Motif III	GRS Domain	Walker A	Walker B	Motif C
Genomovirus	OP549794	LLTYAQ	IHLHV	KAYDYAIK	DVFDVGGYHPNIERVG	GESQLGKTLWAR	AIFDDI	WLAN
Genomovirus	OP549795	LLTYAQ	THYHA	KMFDYATK	RAFDVDGYHPNILRGI	GPTRTGKTSWAR	AVFDDI	WCNN
Genomovirus	OP549796	LVTYAQ	THLHV	KGWEYATK	HIFDVDGYHPNVVPGY	GPTRLGKTVWAR	AVFDDM	WLSN
CRESSV2	OP549818	VFTWNN	EHLQG	QAHTYCKK		GPSGTGKSHFIS	IWLDDV	VTSN
CRESSV2	OP549828	CFTINN	PHLQG	QNHKYCTK		GETGSGKSKSVR	VCIDDF	VTSQ
CRESSV2	OP549833	CFTLNG	KHLQC	QNRRYCIK		GNTGAGKSYLAR	ILLDDF	VTSN
CRESSV2	OP549837	VFTLNN	PHLQG	QAKAYCQK		GDPKAGKSEGAR	IIIEDM	VTSN
CRESSV2	OP549843	TFTINT	PHFQG	QNYDYCSK		GPPRTGKSHKAR	VLIDEL	VTSN
CRESSV6	OP549820	ALTYSN	DHFHA	AWLTYIKK		GESGIGKTNWAK	IIFDDV	FTAN
A	OP549845	FLTINN	PHIQG	ACIIYCTK		GPAGCRKTRTAV	IIIDDF	ITCE
B	OP549827	IYTLNN	PHLQG	DAANYCMK		GPTGVGKTRSVV	TLFDDY	ITCP
B	OP549846	TFTLNN	PHLQG	AAINYCMK		GETGAGKTRYVF	ALLDDF	ITAP
C	OP549840	VFTINN	PHLQG	QAIIYCEK		GPTGSGKSRYAW	AIIDDF	VTCP
D	OP549841	VVTFWC	HHWQA	SNAKYCSK		GSTGRGKSHRTF	VIINEF	VNSS
E	OP549838	CFTLNN	PHLQG	SNREYCSK		GLPGVGKSRRAH	VIIDDF	VTSN
F	OP549819	ILTIPV	HHWQI	AADKYVHK		GGSGLGKTRRAW	VIIDEF	ITSN
G	OP549825	AFTLNN	PHLQG	DSCYYCVK		SRGGAGKSYFAR	IIIIDI	IFAN
G	OP549831	CFTLNN	PHLQG	DNDAYCGG		TEGNVGKSAFTK	LIIWDM	IFSN
G	OP549836	CFTLNN	PHLQG	INLKYCSK		SIGNIGKSAFIK	CIMFDI	IFAN
G	OP549839	IMVLNN	PHLQG	QNDIYCSK		RPGHFGKSQFVK	CVMFDI	CFAN
H	OP549817	DFTIWA	LHYQG	GEPFYVLK		PEGASGKSTLRN	LYVLDL	VFTN
H	OP549832	DFTIKE	LHYQG	DNNFYVMK		TQGNNGKSTLKA	LYILDI	VFMN
I	OP549826	LLTYGK	EHMHV	NAKNYLAK		PVGGSGKTQFAK	GLIFNL	VFAN
J	OP549797	FLTYSQ	FHLHG	RVLHYTQK	RLFDVGIYHPNIGALR	GASRTGKTTWAR	IILDDI	HLCN
J	OP549821	FLTYSQ	IHYHV	RCRHYLRK	DIFDFGGCHAIQPIKN	GPTRLGKTDWAR	LVIDDF	WLCN
J	OP549823	FLTYSQ	IHFHV	NRRHYIRK	NIFDCAGYHPNILPIR	GPTECGKSVWAR	LVLDDM	WICN
J	OP549851	LLTYAQ	QHFHC	NCWEYCTK	RAFDIGQNHPNIKRVG	GPTRTGKTIWAR	LIMDDF	YICN
K	OP549822	FLTYSR	FHLHG	KTLNYIYK	RFLDITGP[DGTVY]HPKLEPVK	GPTKTGKSAWAR	VVFDDI	ILCN
L	OP549824	FLTYPQ	LHLHV	AVMRYCTK		GPTNSGKTALAK	IIYDEA	FTSN
M	OP549834	AYTDFQ	KHIQG	QNFVYCSK		GPTNIGKTQFAI	IVFDDM	FTYN
N	OP549842	FLTYSQ	FHLHC	KCLAYVIK		GESGVGKSTIAT	VVIEDI	ITSN
O	OP549844	FLTYPQ	HHIHA	NVIKYCTK		TKPNLGKTFLFG	VVLDEY	VLSN
P	OP549829	FLTYAQ	PHMHV	AIKKYCMK		GPSNTGKSFWLR	LWADEY	ICSN
P	OP549830	LLTYPQ	PHIHA	RSHKYCQK		GPSNSGKSYWLT	LFSDEY	IVSN
Q	OP549835	FITFPQ	QHLHI	AAIAYITK		GVANVGKTTIIS	AYIDEF	ILSN
S	OP549847	KFTPQK	LHYQC	ALQAYSMK		PSGQVGKTWFGN	CYIIDL	VFAN
S	OP549848	CFTLNN	PHVQG	QARDYCCK		GNTGTGKSTYAR	VVLDEF	AISN
S	OP549849	CFTLNN	HHLQG	QARDYCRK		GPTGCGKTSTAY	VLIDDF	ITTN
S	OP549850	LFTLWL	RHFQS	QNIDYCTK		GSSGTGKTRFAY	VLFDDY	ITSN
S	OP549852	FLTYQS	PHRHV	KCCFYMCK		-	YLVVSL	VVAN
S	OP549853	AMTINN	RHIHV	GWIKYCMK		-	VNYEEL	LYFN
S	OP549854	WLTVNP	FHLHA	DSGQYVFE		-	-	LYTL
S	OP549855	FLTYAQ	PHRHC	NCAKYVLK		GDTGSGKSKYAR	VLLEDV	ITSN
S	OP549856	VFTINY	HHIQG	EARDYALK		GETGRGKTRRAA	VLFDDF	ITSN
S	OP549857	CLTHYG	EHQQA	EARKYCMK		GDTGTGKSHLAH	MIINEF	LTSP

**Table 3:** Summary of the pairwise identity of the Rep amino acid sequences of the cressdnaviruses identified in this study with their top hits. Percentage pairwise identity determined using SDT v1.2 [58].

		Top Rep Hit		
Cluster	Accession	Virus	Rep % Identity	Accession
Genomovirus	OP549794	Gemycircularvirus gemy-ch-rat1	59.7	KR912221
Genomovirus	OP549796	Cybaeus spider-associated circular virus 2 BC_I1644B_C3	60.3	MH545507
Genomovirus	OP549795	Genomoviridae sp. D2_1183	100	MW678959
CRESSV2	OP549818	Diporeia sp.-associated circular virus LM3487	45.8	KC248416
CRESSV2	OP549837	Sewage-associated circular DNA virus-20 NZ-BS3900-2012	48.4	KM821755
CRESSV2	OP549833	Uncultured virus clone CG261	48.2	KY487930
CRESSV2	OP549843	Cressdnaviricota sp. ctdb97	56.3	MH510276
CRESSV2	OP549828	Antarctic circular DNA molecule COCH21_V_94	57.2	MN328284
CRESSV6	OP549820	Circovirus-like genome DCCV-2	47.0	KT149395
A	OP549845	Arizlama virus AZLM_1011	51.3	MW697465
B	OP549827	Uncultured virus clone CG267	42.6	KY487936
B	OP549846	Uncultured virus clone CG267	44.0	KY487936
C	OP549840	Virus sp. D12_1244	63.8	MW678878
D	OP549841	Cressdnaviricota sp. ctcd610	45.4	MH649031
E	OP549838	Sewage-associated circular DNA virus-17 SaCV-17_NZ-BS4236-2012	72.1	KM821752
F	OP549819	Avon-Heathcote Estuary-associated circular virus 26 NZ-2311TU-2012	48.4	KM874359
G	OP549831	Cressdnaviricota sp. ctcd828	54.0	MH649233
G	OP549825	Chicken circovirus 2 strain CCV-2	63.2	MN420497
G	OP549836	Chicken circovirus 4 CCV-4	58.8	MN428454
G	OP549839	Chicken circovirus 4 CCV-4	98.6	MN428454
H	OP549832	Cressdnaviricota sp. ctca156	53.3	MH616996
H	OP549817	Cressdnaviricota sp. ctbb593	48.4	MH648954
I	OP549826	Crucivirus-124 BS_313	42.6	MT263552
J	OP549821	Ancient caribou feces associated virus	53.3	KJ938716
J	OP549823	Sewage-associated circular DNA virus-36 NZ-BS3974-2012	64.9	KM821748
J	OP549851	Genomoviridae sp. 6538_332	36.9	MT309820
J	OP549797	Genomoviridae sp. 6434_400	45.8	MT309859
K	OP549822	Genomoviridae sp. 6538_302	47.6	MT309829
L	OP549824	Uncultured virus clone CG104	40.9	KY487775
M	OP549834	Crucivirus-243 SR3_42497	47.4	MT263577
N	OP549842	Virus sp. D6_821	47.4	MW678874
O	OP549844	Cressdnaviricota sp. ctcj370	58.6	MH617003
P	OP549830	Apis mellifera virus-5 BNH861	85.7	MH973774
P	OP549829	Capybara virus 8_cap1_36	46.2	MK570170
Q	OP549835	Uncultured virus clone CG135	52.0	KY487806
Singleton	OP549847	McMurdo Ice Shelf pond-associated circular DNA virus-8 alg49-57	35.4	KJ547653
Singleton	OP549848	Red panda feces-associated circular DNA virus 7 Rpf101envir01-8	39.1	MZ556225
Singleton	OP549849	Dipodfec virus UA04Rod_4537	41.4	OM869597
Singleton	OP549850	False black widow spider-associated circular virus 1 BC_I1659B_H2	41.4	MH545542
Singleton	OP549852	Sewage-associated circular DNA virus-13 NZ-BS4044-2012	34.6	KJ547624
Singleton	OP549853	Cressdnaviricota sp. Miresoil virus 251	26.4	OM154576
Singleton	OP549854	Cressdnaviricota sp. ctcc233	34.2	MH617714
Singleton	OP549855	Cressdnaviricota sp. Miresoil virus 418	33.9	OM154411
Singleton	OP549856	Circovirus sp. panda384	37.5	MZ556112
Singleton	OP549857	Avon-Heathcote Estuary-associated circular virus 19 NZ-4942GA-2012	29.9	KM874347

**Table 4:** Summary of the pairwise identity of the MCP amino acid sequences of the microviruses identified in this study with their top hits. Percentage pairwise identity determined using SDT v1.2 [58].

		Top MCP Hit		
Subfamily/Clade	Accession	Virus	MCP % Identity	Accession
Unclassified	OP549798	Tortoise microvirus 72	65.4%	MK765625
Alphavirinae-clade	OP549799	Microviridae sp. ctcf586	38.1%	MH617122
Alphavirinae-clade	OP549800	Microvirus sp. BS1_235,	37.3%	MT310011
Alphavirinae-clade	OP549801	Microviridae sp. Flamingo05	58.2%	MG883728
*Gokushovirinae*	OP549802	Microviridae sp. ctcf650	48.0%	MH62292
*Gokushovirinae*	OP549803	Apis mellifera associated microvirus 28 INH_SP_214	71.2%	MH992195
Unclassified	OP549804	Microvirus sp. 1712115_732	71.4%	MT310269
*Gokushovirinae*	OP549805	Microvirus sp. 6433_52	70.0%	MT310113
Pichovirinae-clade	OP549806	Microviridae sp. ctba71	59.9%	MH616766
Unclassified	OP549807	Apis mellifera associated microvirus 42 INH_SP_292	67.4%	MH992217
Unclassified	OP549808	Microvirus sp. 1712115_732	71.9%	MT310269
Gokushovirinae	OP549809	Arizlama microvirus AZLM_259	72.3%	MW697690
Pichovirinae-clade	OP549810	Microviridae sp. SD_MC_53	51.9%	MH572461
*Gokushovirinae*	OP549811	Microviridae sp. ctsGZ299	76.4%	MW202558
*Gokushovirinae*	OP549812	Microviridae sp. ctgc091	43.9%	MH617728
*Gokushovirinae*	OP549813	Arizlama microvirus AZLM_329	65.6%	MW697640
*Gokushovirinae*	OP549814	Microvirus sp. BS1_385	77.1%	MT309971
Pichovirinae-clade	OP549815	Microviridae sp. ctcf880	56.40%	MH617497
Pichovirinae-clade	OP549816	Microviridae sp. ctcf880	56.6%	MH617497

## Data Availability

Short read data are available at NCBI SRA under BioProject: PRJNA880751; BioSample: SAMN30871507; Sequence Read Archive: SRR21617928 and all viral genome sequences have been deposited in GenBank under accession numbers OP549794–OP549857.
